# Impact of supplemental anesthesia in preterm infants undergoing inguinal hernia repair under spinal anesthesia: A retrospective analysis

**DOI:** 10.1007/s00101-022-01199-4

**Published:** 2022-09-19

**Authors:** Benedikt Hermann Siegler, Martha Dudek, Thomas Müller, Markus Kessler, Patrick Günther, Marcel Hochreiter, Markus Alexander Weigand

**Affiliations:** 1 Department of Anesthesiology, Heidelberg University Hospital, Im Neuenheimer Feld 420, 69120 Heidelberg, Germany; 2 Division of Pediatric Surgery, Department of Surgery, Heidelberg University Hospital, Im Neuenheimer Feld 430, 69120 Heidelberg, Germany; 3 Clinic for Anesthesiology and Intensive Care, Essen University Hospital, Hufelandstraße 55, 45147 Essen, Germany

**Keywords:** Safety, Surgery, Prematurity, Adverse events, Apnea, Sicherheit, Operation, Frühgeburtlichkeit, Unerwünschte Ereignisse, Apnoe

## Abstract

**Background:**

In preterm infants, spinal anesthesia (SpA) is recognized as an alternative to general anesthesia for inguinal hernia repair (IHR); however, some patients require supplemental anesthesia during surgery. The purpose of this study was to investigate the frequency and impact of supplemental anesthesia on perioperative care and adverse respiratory and hemodynamic events.

**Methods:**

A retrospective study of preterm infants undergoing IHR at Heidelberg University Hospital within the first year of life between 2009 and 2018 was carried out.

**Results:**

In total, 230 patients (255 surgeries) were investigated. Among 189 procedures completed using SpA 24 patients received supplemental anesthesia. Reasons for supplemental anesthesia included loss of anesthetic effect, returning motor response, and respiratory complications. Compared to SpA alone, no differences were found concerning hemodynamic parameters; however, patients requiring supplemental anesthesia displayed higher rates of postoperative oxygen supplementation and unexpected admission to the intensive care unit. The rate of perioperative apnea was 2.7%. Apneic events exclusively occurred after supplemental anesthesia. Bilateral IHR and duration of surgery were associated with the need for supplemental anesthesia.

**Conclusion:**

Whereas SpA might be favorable when compared to general anesthesia for IHR, the data indicate that particular caution is required in patients receiving supplemental anesthesia due to the possible risk for adverse respiratory events.

## Abstract (de)

**Hintergrund:**

Zur Durchführung einer inguinalen Herniotomie bei Frühgeborenen wird die Spinalanästhesie (SpA) gegenüber der Allgemeinanästhesie als vorteilhaft angesehen. Allerdings benötigen einige Patienten während der Operation ergänzende Medikamentengaben. Ziel dieser Studie ist es, die Häufigkeit solcher ergänzenden Medikamentengaben und deren Einfluss auf die perioperative Behandlung sowie auf unerwünschte hämodynamische und respiratorische Ereignisse zu untersuchen.

**Methoden:**

In einer retrospektiven Analyse des Zeitraums 2009–2018 wurden frühgeborene Patienten analysiert, die innerhalb ihres ersten Lebensjahres am Universitätsklinikum Heidelberg eine inguinale Herniotomie erhalten haben.

**Ergebnisse:**

Insgesamt wurden 230 Patienten (255 Operationen) untersucht. Im Rahmen der 189 Eingriffe, die in SpA durchgeführt wurden, erhielten 24 Patienten ergänzende Medikamentengaben. Als Gründe dieser Medikamentengaben wurden das Abklingen der Spinalanästhesie, zurückkehrende motorische Reaktionen sowie respiratorische Komplikationen identifiziert. Im Vergleich zu den Patienten, die nach SpA keine ergänzenden Medikamente erhielten, zeigten sich keine Unterschiede bezüglich hämodynamischer Parameter. Allerdings war die ergänzende Medikamentengabe nach SpA mit einer erhöhten Notwendigkeit einer postoperativen Sauerstoffgabe sowie mit einer höheren Rate an unerwarteten Aufnahmen auf die Intensivstation assoziiert. Die Häufigkeit postoperativer Apnoe-Ereignisse betrug 2,7 %. Diese traten ausschließlich nach Gabe zusätzlicher Medikamente auf. Als prädiktive Faktoren für die Notwendigkeit einer ergänzenden Medikamentengabe wurden die bilaterale Herniotomie und die Dauer des Eingriffs identifiziert.

**Schlussfolgerung:**

Obgleich die Spinalanästhesie gegenüber der Allgemeinanästhesie zur Durchführung einer inguinalen Herniotomie von Vorteil zu sein scheint, verdeutlichen unsere Ergebnisse, dass eine erhöhte Vigilanz insbesondere bei Patienten mit der Notwendigkeit ergänzender Medikamentengaben geboten ist, da diese mit dem potenziellen Risiko unerwünschter respiratorischer Ereignisse behaftet sind.

## Introduction and background

With incidences ranging between 13% and 30% depending on birth weight, inguinal hernia—persistency of the processus vaginalis with temporary dislocation of intra-abdominal organs—is one of the most prevalent indications for surgery in neonates and young children [9, 21]. Unfortunately, a high proportion of all hernias correspond with prematurity, which is not only the leading risk factor for inguinal hernia but is also a key determinant of infant mortality [11].

It is well known that the risk of anesthesia-related complications rises significantly in neonates and preterm infants [25]. In particular, the high vulnerability to apnea and other cardiorespiratory problems represents a major concern in the anesthetic management of these patients, especially when general anesthesia (GA) is performed [1]. While the frequency of apnea declines with increasing postmenstrual age (PMA) [3], surgical intervention should be performed timely after diagnosis in order to avoid surgical complications, like bowel incarceration or gonadal necrosis [16, 24].

In view of the potential side effects of GA, current evidence supports the use of regional techniques, including spinal anesthesia (SpA), which was found to be significantly less associated with postoperative apnea and other adverse cardiorespiratory events in infants undergoing inguinal hernia repair (IHR) [5, 13]; however, in some cases, the clinical benefits of SpA may be outweighed by the disadvantages, i.e. by loss of anesthetic effect during surgery, returning motor response, or respiratory complications, thereby resulting in additional drug administration or even switching to GA.

So far, little is known about the extent to which supplemental anesthesia affects cardiorespiratory outcomes and morbidity in patients with prior spinal blockade. A subgroup analysis of the *General Anesthesia compared to Spinal anesthesia* study revealed no case of apnea in infants at early age undergoing SpA and receiving short-term additional sedatives or sevoflurane during surgery [4]. Yet importantly, only half of the patients in the GAS study were ex-premature, and the study excluded infants born at less than 26 weeks of gestation or with severe cardiac comorbidities.

Our study’s intention was to analyze the frequency and impact of supplemental anesthesia on intraoperative and postoperative adverse cardiorespiratory events in all preterm infants undergoing IHR with SpA at our institution. In addition, we aimed to identify factors associated with the need for supplemental anesthesia in order to enable better risk stratification in this highly vulnerable patient population.

## Study design and investigation methods

### Ethics and data collection

After obtaining approval from the ethics committee of the medical faculty of the Heidelberg University (approval number S‑283/2017), a retrospective analysis was conducted in accordance with the principles expressed in the 1964 Helsinki Declaration and its later amendments. All IHRs performed at Heidelberg University Hospital in preterm infants from January 2009 to December 2018 were included in the primary data assessment. Due to the retrospective study design, written informed consent was not obtained. Both elective and emergency IHRs were analyzed without excluding any pre-existing patient comorbidities or other conditions. In patients requiring another surgery after their primary discharge or more than 14 days after the first IHR, each procedure was considered as a separate case. Incomplete data sets were excluded from the final analysis.

### Baseline and perioperative characteristics

Evaluated data included gender, gestational age, and birth weight as well as PMA and weight at the time of surgery. Postmenstrual age was defined as the gestational age plus the time between birth and surgery, according to a statement from the Committee on Fetus and Newborn of the American Academy of Pediatrics [6]. Patients’ preanesthesia categorization, according to the physical status classification system of the American Society of Anesthesiologists (ASA), and perinatal comorbidities connected with prematurity as well as details on the surgical procedure (bilateral or single site hernia repair) were recorded.

### Anaesthetic procedure

#### Spinal anesthesia

According to the in-house standard, patients receiving SpA were either held in a lateral or sitting position by the assisting nurse, with attention paid to lumbar kyphosis in order to optimize the puncture conditions. If necessary, patients received a short general anesthesia using sevoflurane via facemask or were briefly calmed with oral glucose to enable optimal puncture conditions. The intrathecal space was primarily accessed at L4–L5 or L5-S1 by median puncture under sterile conditions using 25 mm, 25-gauge, or 26-gauge spinal needles. When cerebrospinal fluid returned, a mixture containing 0.5% isobaric bupivacaine (1 mg per kg bodyweight) and clonidine (1.5 µg per kg bodyweight) was applied. The needle was extracted and the infant was immediately positioned for surgery. Any further movement of the patient (i.e. to place a neutral electrode) was kept to a minimum in order to avoid extensive cranial spreading of the local anesthetic. Before surgery began, the success of SpA (loss of autonomic or motor response to external stimuli) was tested via gentle tactile stimulation of the thigh with a forceps.

#### Supplemental anesthesia

Supplemental anesthesia was defined as additional administration of intravenous or inhalative anesthetics throughout surgery after an initially successful spinal puncture. Due to the retrospective character of this study, the anesthetic procedure in patients requiring supplemental anesthesia, including the type of anesthetics or airway devices, was not predefined.

### Perioperative care, cardiorespiratory outcomes, and morbidity

Time from the end of spinal puncture to first skin incision, duration of surgery (time from first skin incision to last suture), length of stay (LOS) in the recovery room and in the hospital, as well as the rate of admissions to intensive care unit (ICU) were recorded. Any ICU admission not planned before surgery, i.e. due to the occurrence of perioperative adverse events, was termed ‘unexpected’.

All patients were routinely monitored during and after surgery until discharge. Their vital signs were recorded on paper-based forms every 5 min in the theater as well as in the recovery room. Hypotension was defined as any drop of mean arterial pressure below 35 mmHg. Bradycardia was defined as a recorded pulse less than 100 beats per minute. Documentation of perioperative care was further screened to investigate the use of fluid boluses or vasoactive medication as well as the rate of respiratory adverse events. Patients were closely observed by the caretaking medical staff familiar with the detection and current definition of apnea. Postoperative need for oxygen supplementation was defined as any need for noninvasive oxygen delivery, i.e. via nasal oxygen cannula in order to maintain an oxygen saturation above 92%.

### Statistical analysis

Statistical analysis was performed using GraphPad Prism (Version 9.0 f, GraphPad Software, San Diego, CA, USA). For descriptive statistics, continuous data are presented as mean ± standard deviation (SD), or as number (*n*) and percentage in case of categorical variables. Comparisons between groups were conducted using the Mann-Whitney *U* test for continuous, and Fisher’s exact test for categorical data. To identify possible risk factors for supplemental anesthesia in patients undergoing SpA, a binary, multivariate, logistic regression model was calculated. *P* values < 0.05 were considered statistically significant.

## Results

### Patient characteristics

**Fig. 1:**
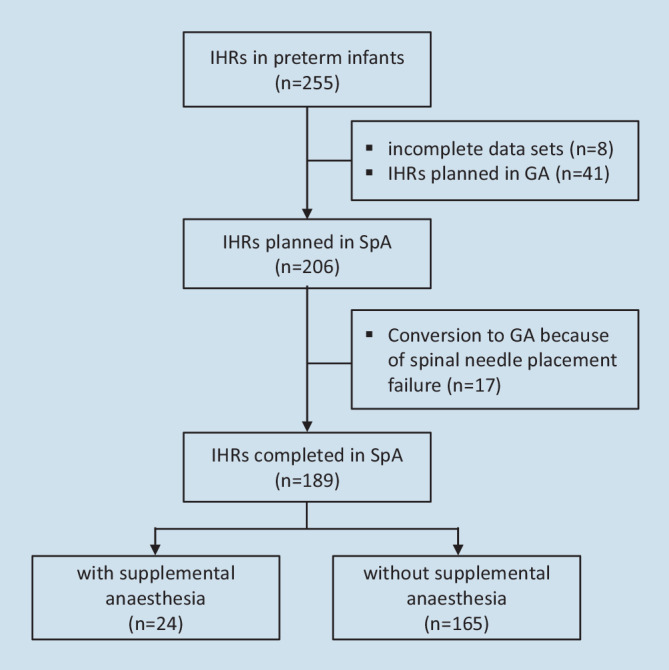
Study population. *IHRs* inguinal hernia repairs, *GA* general anesthesia, *SpA* spinal anesthesia

In total, 230 preterm infants underwent 255 surgeries for IHR at our institution from January 2009 to December 2018 with 8 IHRs being excluded from final analysis due to incomplete data sets. Among the remaining 247 IHRs, 41 were performed with planned GA. While SpA was attempted for 206 procedures, spinal needle placement failure occurred in 17 patients (8%) who subsequently received GA. Overall, 189 IHRs were completed using SpA (Fig. 1). Among these, 28 (15%) were briefly calmed/sedated to enable successful spinal puncture. The mean duration of surgery was 37 min, ranging from 15 to 149 min. Most IHRs were unilateral (94%). Table 1 summarizes baseline demographics and clinical characteristics of the investigated population.

**Table 1:** Demographics and clinical characteristics

Variables	Patients receiving SpA			*p*-value
	Total (*n* = 189)	Without supplemental anesthesia (*n* = 165)	With supplemental anesthesia (*n* = 24)	
*M:F*	163:26	141:24	22:2	0.54
*Gestational age* (weeks)	29 ± 4	29 ± 4	29 ± 4	0.62
*PMA at surgery* (weeks)	40 ± 4	41 ± 4	39 ± 3	0.14
*Birth weight* (g)	1206 ± 669	1205 ± 665	1214 ± 692	0.94
*Weight at surgery* (g)	2892 ± 824	2927 ± 849	2651 ± 573	0.13
*Comorbidities* (*n*)				
Apnoea and bradycardia	150 (79)	132 (80)	18 (75)	0.59
Respiratory insufficiency	144 (76)	125 (76)	19 (79)	0.80
Cardiac malformation	75 (40)	64 (39)	11 (46)	0.51
Neonatal infection	82 (43)	73 (44)	9 (38)	0.66
Ventricular haemorrhage	28 (15)	23 (14)	5 (21)	0.36
*ASA classification* (*n*)				
ASA 1	35 (19)	29 (18)	6 (25)	0.40
ASA 2	96 (51)	89 (54)	7 (29)	**0.03**
ASA ≥ 3	58 (31)	47 (28)	11 (46)	0.10
*Sedation for spinal puncture* (*n*)	28 (15)	25 (15)	3 (13)	> 0.99
*Side of IHR* (*n*)				
Bilateral	11 (5.8)	7 (4.2)	4 (17)	**0.04**
Unilateral	178 (94)	158 (96)	20 (83)	**0.04**
Right side	95 (50)	83 (50)	12 (50)	> 0.99
Left side	83 (44)	75 (45)	8 (33)	0.28
*Time from end of spinal puncture to skin incision* (min)	9.8 ± 6.0	9.7 ± 5.9	11 ± 6.8	0.40
*Duration of surgery* (min)				
Total	37 ± 17	36 ± 14	44 ± 29	0.56
Bilateral IHR	44 ± 11	40 ± 8.6	52 ± 11	0.17
Unilateral IHR	38 ± 17	36 ± 14	42 ± 31	0.82
*Hospital LOS* (days)	68 ± 55	67 ± 55	72 ± 55	0.52

Data are presented as mean ± SD or *n* (%). *P* values <0.05 are marked as bold numbers.

*ASA* American Society of Anesthesiologists,* IHR* inguinal hernia repair,* LOS* length of stay,* PMA* postmenstrual age, *SpA* spinal anesthesia

### Supplemental anesthesia

Patients in 24 out of 189 IHRs (13%) performed with SpA received supplemental anesthesia during surgery (Fig. 1). The rate of sevoflurane use to enable spinal puncture was similar in patients with or without supplemental anesthesia (OR 1.3, 95% CI 0.36–4.2, *p* > 0.99). Twenty-three patients required supplemental anesthesia due to returning motor or autonomic responses, while one patient was intubated due to intraoperative apnea and impeded mask ventilation. Overall, four patients were orally intubated, while a laryngeal mask was inserted in eight cases. Twelve patients were sedated with sevoflurane under supported spontaneous breathing via facemask. Table 2 lists details on the anesthetics used. Aside from one patient, who was directly transferred to the ICU, all patients were successfully weaned and extubated after surgery.

**Table 2:** Anesthetics used in patients receiving supplemental anesthesia

Anaesthetics	Airway device		
	Facemask^a^ (*n* = 12)	Laryngeal mask (*n* = 8)	Oral intubation (*n* = 4)
*Opioids* [*n*]			
Alfentanil	0 (0.0)	5 (63)	4 (100)
*Hypnotics* [*n*]			
Sevoflurane	12 (100)	4 (50)	1 (25)
Propofol	0 (0.0)	2 (25)	1 (25)
Thiopental	0 (0.0)	1 (13)	2 (50)
Etomidate	0 (0.0)	1 (13)	0 (0.0)
*Muscle relaxants* [*n*]			
Mivacurium	0 (0.0)	0 (0.0)	3 (75)
Cis-atracurium	0 (0.0)	0 (0.0)	1 (25)

Data are presented as *n* (%)

^a^Assisted spontaneous breathing

### Impact of supplemental anesthesia on hemodynamics, adverse cardiorespiratory events, and perioperative care

Incidence and treatment of adverse cardiorespiratory events, as well as data for perioperative care, are provided in Tables 3 and 4, respectively. Neither the frequency of bradycardia and hypotension nor the need for cardiocirculatory medication or fluid boluses differed between patients with or without supplemental anesthesia. A higher proportion of patients receiving supplemental anesthesia required oxygen application via nasal cannula in the recovery room (OR 2.9, 95% CI 1.2–7.0, *p* = 0.03). The rate of postoperative oxygen requirement significantly differed comparing the used airway devices and was lower in patients with bag-mask ventilation versus patients with laryngeal mask (OR 0.0, 95% CI 0.00–0.12, *p* < 0.0001) and in patients with bag-mask ventilation versus patients with oral intubation (OR 0.03, 95% CI 0.00–0.49, *p* = 0.03).

**Table 3:** Perioperative adverse cardiorespiratory events

Variables	Patients receiving SpA		*p*-value
	Without supplemental anesthesia (*n* = 165)	With supplemental anesthesia (*n* = 24)	
**Cardiovascular adverse events**			
*Bradycardia*			
During surgery	7 (4.2)	2 (8.3)	> 0.99
In the recovery room	2 (1.2)	0 (0.0)	> 0.99
*Hypotension*			
During surgery	55 (33)	8 (33)	> 0.99
In the recovery room	1 (0.6)	0 (0.0)	> 0.99
**Respiratory adverse events**			
Bronchospasm	0 (0.0)	0 (0.0)	> 0.99
Postoperative apnoea	0 (0.0)	5 (21)	**<** **0.0001**
Postoperative need for oxygen supplementation	28 (17)	9 (38)	**0.03**
**Need for cardiocirculatory medication**			
Cafedrine/theodrenaline	22 (13)	3 (13)	0.43
Norepinephrine	1 (0.6)	0 (0.0)	> 0.99
Atropine	7 (4.2)	2 (8.3)	> 0.99
**Administration of fluid boli**			
Balanced crystalloids	47 (28)	6 (25)	0.81
Albumin 5%	1 (0.6)	0 (0.0)	> 0.99

Data are presented as mean ± SD or *n* (%). *P* values <0.05 are marked as bold numbers.

*SpA* spinal anesthesia

**Table 4:** Postoperative care

Variables	Patients receiving SpA		*p*-value
	Without supplemental anesthesia (*n* = 165)	With supplemental anesthesia (*n* = 24)	
*LOS in the recovery room* [min]	79 ± 40	80 ± 38	0.92
*Postoperative ICU admission*			
Total	5 (3.0)	4 (17)	**0.02**
De novo	1 (0.6)	2 (8.3)	**0.04**

Data are presented as mean ± SD or *n* (%). *P* values <0.05 are marked as bold numbers.

*SpA* spinal anesthesia, *ICU* intensive care unit

In total, postoperative apnea was recorded in five cases (2.7%). Notably, apneic events exclusively occurred immediately after surgery or in the recovery room and in patients who received supplemental anesthesia (*p* < 0.0001). None of these five patients was exposed to brief sedation for spinal puncture. Three patients with subsequent apnoea received sevoflurane, two received alfentanil, hypnotics and muscle relaxants.

There were no observed late events of apnea (after discharge from the recovery room). The LOS in the recovery room was comparable between both groups (79 ± 40 min versus 80 ± 38 min, *p* = 0.92). Both the total amount as well as the number of unexpected admissions to the ICU were significantly higher when supplemental anesthesia was administered during surgery (OR 6.4, 95% CI 1.8–24, *p* = 0.02 and OR 15, 95% CI 1.6–217, *p* = 0.04, respectively).

### Predisposing factors associated with the need for supplemental anesthesia

There were no differences in gender, gestational age, PMA at surgery, birth weight, and weight at the time of surgery or comorbid conditions between the patients undergoing IHR in SpA with or without supplemental anesthesia. There was a higher proportion of bilateral IHRs in patients receiving supplemental anesthesia (OR 4.5, 95% CI 1.4–16, *p* = 0.04, Table 1). The odds for supplemental anesthesia were significantly lower in patients classified as ASA 1 (OR 0.22, 95% CI 0.05–0.88, *p* = 0.03). Multivariate logistic regression analysis identified bilateral IHR (OR 6.0, 95% CI 1.1–30, *p* = 0.03) and duration of surgery (OR 1.0, 95% CI 1.0–1.1, *p* = 0.04) as predictors for the need for supplemental anesthesia (Table 5).

**Table 5:** Multivariate analysis using logistic regression for potential risk factors for the event ‘supplemental anesthesia’

Odds ratio estimates and Wald confidence intervals			
Effect	Estimate	95% CI	*p*-value
*Female sex*	0.88	0.12–4.0	0.88
*Gestational age*	0.87	0.62–1.2	0.40
*PMA at surgery*	0.97	0.73–1.3	0.86
*Birth weight*	1.0	0.99–1.0	0.51
*Weight at surgery*	1.0	0.99–1.0	0.16
*Comorbidities*			
Apnea and bradycardia	0.28	0.06–1.3	0.10
Respiratory insufficiency	1.8	0.41–8.2	0.46
Cardiac malformation	0.76	0.21–2.7	0.66
Neonatal infection	0.39	0.10–1.3	0.15
Ventricular hemorrhage	1.2	0.27–4.3	0.81
*ASA classification*			
ASA 1	0.22	0.05–0.88	**0.03**
ASA 2	0.40	0.07–2.2	0.28
ASA ≥ 3	3.1	0.26–35	0.35
*Sedation for spinal puncture*	0.71	0.12–3.0	0.66
*Bilateral IHR*	6.0	1.1–30	**0.03**
*Time from end of spinal puncture to skin incision*	1.0	0.95–1.1	0.39
*Duration of surgery*	1.0	1.0–1.1	**0.03**

*ASA* American Society of Anesthesiologists, *CI* confidence interval, *IHR* inguinal hernia repair, *PMA* postmenstrual age. *P* values <0.05 are marked as bold values.

## Discussion

With respect to the increase in cardiorespiratory morbidity associated with GA, current evidence supports the use of neuraxial techniques, including SpA, in neonates undergoing IHR [5, 13]. Clinical outcomes, including postoperative apnea, have been widely investigated in neonates after GA [1, 3, 10, 18, 20] or by comparing general versus regional anesthesia [2, 4, 15, 23, 26, 27]. While in general, neuraxial techniques can be considered safe (i.e. as shown by Hoelzle et al. [12]), studies focusing on the impact of supplemental anesthetics in premature patients receiving SpA are missing. To close this gap, we retrospectively assessed intraoperative and postoperative adverse events in pre-term and early term infants undergoing IHR at our institution in SpA with or without SA throughout the intervention.

Among 206 procedures, 17 patients (8%) were converted to GA before surgery began, which is in line with the SpA failure rate of 7.5% described in a recent meta-analysis [5]. In 165 out of 206 cases (80%), SpA was sufficient on its own to complete the surgical intervention, while 24 patients (13%) required supplemental anesthesia. Both SpA failure rate as well as the proportion of patients requiring supplemental anesthesia were slightly higher than reported in a nested cohort study within the GAS trial by Frawley et al. [7]. In their study, failure rate was 5.9% and 7.2% of patients required supplemental anesthesia. However importantly, patients in our cohort were younger (mean PMA 40 versus 45 weeks) and had a lower mean weight (2.8 versus 4.1 kg) at the time of surgery, which might have contributed to the lower SpA success rates in the present study.

One of the largest retrospective reviews on SpA in preterm infants undergoing IHR using the same inclusion criteria considered here was published by Frumiento et al. [8]. While the mean duration of surgery in their study was comparable to ours, we found a lower rate of required supplemental anesthesia (13% versus 21%), which might be explained by the use of different local anesthetics and the intrathecal clonidine administration in our patients to lengthen spinal blockade. Nevertheless, using a multivariate logistic regression, we identified bilateral IHR and a longer duration of surgery as being associated with the need for supplemental anesthesia.

Concerning hemodynamic parameters in our patients, the frequency of hypotension or bradycardia did not vary between infants with or without supplemental anesthesia and there was no difference according to perioperative cardiovascular medication. However, importantly, our analysis revealed an association between administering supplemental anesthetics following SpA and postoperative respiratory morbidity. Despite a higher proportion of patients requiring oxygen supplementation in the recovery room, we found that the recorded events of apnea exclusively occurred during the early postoperative period. These apneic events were restricted to patients with prior exposure to supplemental anesthesia during surgery.

As another important difference to Frumiento et al. [8], sevoflurane was used in most patients requiring supplemental anesthesia, followed by intravenous anesthetics including propofol, which has also been used by others to pacify infants during surgery [14, 28]. None of our patients received ketamine, which has been associated with postoperative apnea and bradycardia when used as adjunct sedation for SpA [26]. Interestingly, our analysis revealed that apneic events not only occurred in patients exposed to intravenous anesthetics for supplemental anesthesia, but also, and even more frequently, in infants with supported spontaneous breathing via facemask under sevoflurane sedation.

With the introduction of volatile anesthetics in clinical practice, several studies investigated potential risks associated with sevoflurane administration in neonates undergoing IHR [19, 22]. Murphy et al. identified sevoflurane as an anesthetic risk factor for postoperative apnea in a retrospective chart review of 126 premature infants undergoing IHR in combined caudal and general anesthesia [18]. Another small, randomized controlled study of 24 patients revealed higher rates of apnea in patients receiving sevoflurane compared to those undergoing surgery with SpA [27].

The association between sevoflurane sedation alone and adverse respiratory events was investigated in a current analysis by Lei et al. of pediatric patients undergoing magnetic resonance imaging scans. Remarkably, the apnea rate of 2.4% in preterm infants was tenfold higher compared with term infants [17]. In contrast, the GAS study revealed no case of apnea in patients with SpA receiving short-term additional sedatives or sevoflurane throughout surgery [4]. Yet importantly, only 50% of the patients in the GAS study were ex-premature, and it excluded infants born at less than 26 weeks of gestation or with severe cardiac comorbidities [4]. In contrast, in our analysis, two out of three apneic events in patients receiving supplemental anesthesia with sevoflurane occurred in infants with a gestational age of 23 weeks. Both had a history of congenital heart disease. As a result, we assume that the risk of adverse respiratory events following sedation, including sevoflurane, increases in patients with low gestational age and relevant comorbidities. This is further strengthened by a significant increase in unexpected admissions to the ICU in patients receiving supplemental anesthesia. However, conversely, with respect to the absence of apneic events in patients undergoing spinal blockade without supplemental anesthesia, our data also show that SpA can be considered a safe method for early and preterm infants undergoing IHR.

We acknowledge several limitations. First, our study is a single-center retrospective analysis. Regardless, this design enabled us to compare the anesthetic technique and perioperative management of a relatively high number of preterm infants undergoing SpA. Second, since perioperative anesthetic documentation at our institution was mainly paper-based during the study period, automated data extraction was not feasible. Third, the patients in our study were not routinely monitored with impedance pneumography as the most sensitive technique for apnea detection. However, trained medical staff familiar with the detection of apnea closely monitored and observed all of the patients involved. In addition, since we enable parents to visit their children as soon as possible in the recovery room and encourage early breastfeeding, we might also have prevented some events of apnea through natural tactile stimulation in this high-risk patient collective.

## Conclusion

Overall, our study confirms that SpA is a safe anesthetic technique for early and preterm infants undergoing IHR, a population at risk of adverse cardiorespiratory events. However, our analysis revealed bilateral IHR and duration of surgery as predictors for the need for supplemental anesthesia. While administration of supplemental anesthetics does not impede hemodynamic parameters, our data indicate that particular vigilance concerning postoperative apnea is required in patients receiving supplemental anesthesia, including sevoflurane sedation throughout surgery.
